# An Experimental Enquiry into the Physiology of Cutaneous Absorption, and Its Application to Therapeutics, &c.

**Published:** 1838-10

**Authors:** 


### Art. IV.

*An Experimental Enquiry into the Physiology of Cutaneous Absorption, and its Application to Therapeutics, &c.* By W. H. Madden, m.d.—*Edinburgh*, 1838. 8vo. pp. 151.

It cannot be doubted that the rewards which have, within the last few years, been offered at some of our medical schools and universities for proficiency in the various branches of medical study, have, among a certain class of their students, been productive of most beneficial results. The volume before us is one among those results. If we turn through a pile of the theses which, either in accordance with the rules of the university or the vanity of early authorship, have been given to the world, we find, with an occasional exception, productions which clearly proclaim the bare necessity or vanity which conceived and gave them birth. The present essay, "that to which the Medical Faculty of the University of Edinburgh awarded a gold medal, at the graduation on the 1st of August, 1837," is a very gratifying evidence of the discretion of the body which established the reward. It is written by one who has acquired a full knowledge of the subject of which he treats; in an excellent style; with a clear and systematic arrangement; and with a cautiousness of expression towards those whose opinions he has found reason to oppose, with which, in a young author, it is extremely agreeable to meet. There has been long known a large class of facts, both occurring in the healthy and unhealthy states of the body, which tend strongly to favour the idea of an absorbent quality in the skin. Such are the effects of the local application of medicine; the relief afforded to great thirst by external moisture; the apparently nutritive effect of baths occasionally employed when other means of conveying nourishment into the system were inapplicable, and the like: and numerous direct experiments, such as those of Dr. Young and many other physiologists, have afforded positive evidence of a kind scarcely to be contradicted. The contradiction has, however, been given; and we, therefore, consider experiments as valuable, which, although they contain nothing in themselves very novel, and lead to no new conclusions, add a mass of new facts to one side of the question; and determine, with all the evidence which the case admits, that which is the true side. That Dr. Madden has done so much, we think will be admitted; and he has, besides, examined with great care, and with much success, the experiments on which inferences opposite to those which he has himself been led to adopt repose; showing that some of them, at least, lead to conclusions contrary to those which their authors derived from them. In the following pages, our object will mainly be to give an analysis of the essay before us; the opinions which it contains being so much in accordance with our own that we have scarcely an objection to offer to them.

The thesis is divided into four parts: 1, The Anatomy of the Skin; 2, Absorption from its Uninjured Surface; 3, Endermic Absorption; 4, The Agents by which Absorption is Effected, and the Application of the Subject to Therapeutics.

In the first chapter of the First Part, it has been the object of the author to point out the analogy of structure of the skin of the lower classes of animals with that of man; from whence he infers that "the striking similarity which we have seen to exist in these organs, throughout the animal kingdom, must be a powerful collateral evidence in favour of cutaneous absorption, since even its most determined opponents are compelled to admit that its existence in the lower tribes is unquestionable." Whatever argument can be founded on analogy of structure we agree with Dr. Madden in considering legitimate in the case before us.

Chapter ii. is devoted to the *Anatomy of the Dermis.* We greatly regret that, in an essay of such considerable merit as the one before us, we should have to charge its author with unfair dealing towards ourselves. This chapter is mainly founded on the researches of Breschet and Gurlt. In our second volume, after a very careful examination of their separate works, as well as of the discoveries of others in the same field of enquiry, we arrived at certain conclusions which we think have been confirmed by subsequent experience. It may be considered as asserting too much to say that our conclusions have been adopted without any acknowledgment by Dr. Madden; but such an inference is warranted when we find that, without the slightest allusion to the source whence they have been derived, our quotations have been appropriated, and in the very form in which we had abridged them from the original works.*

By his own examination, the author has satisfied himself of the existence of the sudorific apparatus described by Breschet and Gurlt. He is not satisfied as to the existence of vessels in the epidermis, from the extreme difficulty of coming to any certain conclusions from the appearance of this substance beneath the microscope. He does not appear to be aware of the existence of injected preparations of the epidermis; and if they are not to be regarded as proofs of its vascularity, we know not on what evidence the vascularity of mucous membranes can rest. To these preparations we have alluded, and from personal observation, in the article already referred to. Some recent examinations of cuticle by Dr. Henle have disclosed its intimate structure, apparently, with great minuteness. These will be new to many of our readers.

"The scales in the external parts of the cuticle have a most irregular form, being subrotund, angular, polyedral, and frequently with imperfect or lacerated margins, but all with flat surfaces, frequently bent, and so thin that the microscope discovers innumerable quantities of them, in the smallest particle which the naked eye can see. In these it is rarely possible to discover any traces of a central spot. But from considering the manner in which the cuticle grows, from examining this membrane as it exists in the embryo, and attending to its structure in the adult, where in close apposition to the skin, or where naturally thin and delicate, as on the glans penis and prepuce, Dr. Henle has been led to believe that such are not the regular forms. For in all these situations, he found the scales thicker, and bearing the appearance of cells, with straight margins, generally of a pentagonal form and all provided with a central nucleus. This nucleus, which is in the centre of each cell, has a smooth or slightly granulated surface, is subrotund or oval, sometimes appears to contain in it lesser granules, and is thicker than the cell, so that it projects on each side. The so called rete Malpighii consists of a similar structure, with this exception, that the nuclei of the same size as in other parts, occupy almost the whole of the cells." . . . [The investigations of Dr. Sharpey and of the author confirm the above statements.] . . . "In the

* In confirmation of what we have above said, we refer the reader to pages 17 and 25 of Dr. Madden's Thesis, and to Vol. II. pages 435, 436, 441, of the British and Foreign Medical Review.

epithelium of mucous membranes, which, as will be seen immediately, possesses a structure closely resembling that of the epidermis, the cells are united by means of a delicate net-work of some substance, the precise nature of which is as yet unknown, and we may therefore reasonably conclude that the same means of connexion also exist in the parts we are now considering." (p. 28-29.)

In favour of the cuticle being an organized substance, the author alludes to its definite structure, as above shown; to its differences in different parts, according to the specific purposes it is destined to fulfil; to the fact of its being the seat of a disease to which Dr. Wallace applied the term *morulus*, and in which the true skin is unaltered; and to its resisting chemical reagents which act upon it immediately that it is separated from its connexion with the body. Its permeability by fluids is regarded as established by the various recorded facts of the transmission of fluids, such as ink, spirit of turpentine, &c.; and by what is observed when the feet have been for some time soaked in warm water, the thick cuticle of the sole becoming whitened and opaque, and allowing a quantity of fluid to exude if pressure is then made upon it. The analogy between skin and mucous membranes has generally been maintained.

"In the conjunctiva and the mucous membrane lining the mouth and œsophagus the scales and cells present the exact resemblance of those which we have already described as existing in the skin. At the cardiac orifice of the stomach, these cells disappear, and are replaced by cylindrical bodies. They again reappear in the body of the stomach, and are finally lost at the pylorus: the whole remaining extent of the intestinal mucous membrane being covered by cylindrical or cuneiform bodies, united by their sides and fixed to the subjacent tissues by their apices. These cylinders are connected by a net-work of some unknown substance, and have often, though not always, the central nucleus. In the air passages, the vibratory cilia are placed upon analogous cylinders, an additional proof, if such were needed, that they are truly organized, for although it has appeared probable to many that a layer of inorganic matter should cover the surface of a living tissue, few, I apprehend, would be inclined to hold the same opinion with regard to one which was interposed between two living organized structures, and such are both the mucous membrane and the vibratory cilia." (p. 34.)

The analogy shown to exist between the structure of the skin and mucous membranes must be regarded as in some degree justifying the inference of similarity of function.

The second division of Dr. Madden's essay is devoted to the consideration of *Absorption from the Uninjured Skin.* In Chap. I., which treats of "*Absorption in the Bath,*" are considered the effects of baths, both general and local, of pure water and of water containing nutrient matters. The importance of this enquiry is justly regarded as enhanced by the fact that the opponents of cutaneous absorption have regarded the experiments performed in the warm bath as the most conclusive in favour of their opinions. We shall pass over the author's account of early opinions and experiments on the subject, as, from not having guarded against certain sources of fallacy, and from not having conducted them with sufficient minuteness, experimenters have drawn inferences which have been rendered inconclusive by subsequent experience. Some recent experiments of Collard de Martigny, in which all sources of fallacy appear to have been guarded against, tend strongly to establish the fact of cutaneous absorption.

"By means of a very sensible balance, capable of turning with a quarter of a grain, he carefully weighed a handkerchief. Removing this, he carefully placed in each scale a china vessel of the same diameter, established an equilibrium between them, and then poured into both an equal quantity of tepid water. One of the vessels was then removed from the scale, and replaced by weights equal to the other, and thus the quantity of water employed was determined. These circumstances were all carefully noted. The weight of the handkerchief and of the water being thus known, and a perfect equilibrium established between the two vessels, he plunged his arms up to the elbows in one, leaving the other untouched, remained thus for half an hour, then dried his arms with the handkerchief and weighed it immediately. Having now replaced the vessels under precisely the same circumstances as before, he found the one in which his arms had been immersed, considerably lighter, and accordingly added weight to restore the equilibrium. The experiment was performed six hours and a half after taking food, the air was cold and dry, the day serene, and the temperature of the water, 74° F. It gave the following results:

| | | |
|---|---|---|
| "Weight of the handkerchief . . . . . . | before, | ℥ij. + gr. xiv. |
| | after, | ℥ij. + gr. xl. |
| Increase . . . . . . . | | gr. xxvi. |
| Weight of each vessel full of water in equilibrium, | before, | ℥xij. + ʒiv. + gr. xxiv. |
| Weight of lightest, . . . . . . . | after | ℥xij. + ʒij. + gr. lxvss. |
| Total loss . . . . . . . . . | | gr. 104½ |
| Water absorbed by handkerchief . . . . . . . . | | gr. 26 |
| Absolute loss . . . . . . . . . | | gr. 78½." |

(p. 40.)

The following experiment we select from among others by Collard, tending to establish the fact of cutaneous absorption from the hand:

"He took a tube of glass, bent like a syphon, and widened into a funnel at one extremity; some mercury was placed in the bend, and the limb near the funnel was filled with water. The palm of the hand was then applied to it, and retained there for the space of two hours, at the expiration of which period the column of mercury had risen considerably towards the hand, thus clearly indicating the absorption of some part of the water. Milk, tried in the same way, was absorbed less quickly, soup more rapidly than water." (p. 41.)

Supposing these experiments to be strictly accurate, we can see no other inference to be derived from them, than that an absorbing power was possessed by those parts of the skin with which the fluid came in contact. They appear to have been well devised, carefully and minutely conducted, and with a knowledge and an avoidance of the ordinary sources of mistake. The experiments which Dr. Madden has himself performed on this part of the subject are justly regarded by him as strongly corroborating the above-mentioned testimony; but, as they add nothing to the strength of this, and as they are objectionable (and the first four of them in particular) on the ground that there was no absolute certainty that the arm was always immersed to exactly the same degree in the fluid, we shall pass them over, particularly as we must give more at length Dr. Madden's observations on the effect of *general baths.* The ancients appear to have placed considerable reliance on the effects, both of medicinal and nutritive fluids, when applied to the skin; and there are very numerous facts which afford a certain degree of foundation for the justness of this opinion. Various physiologists have laboured, with various success, to establish or to refute the doctrine of absorption from the whole surface: and, among the opponents of the doctrine, Dr. Madden refers especially to Séguin, the results of whose experiments are often referred to as conclusive against cutaneous absorption; but which results he regards as far from justifying such a conclusion. Our author's remarks on this subject are very valuable, and we shall therefore condense them. After thorough washing, Séguin was weighed in the air, and the process was repeated after a certain interval, the whole loss of weight being divided by the number of minutes during which the experiment lasted, the quotient indicated the mean loss for every minute. Immediately afterwards he entered the bath, and, at the end of three or four hours, was again weighed, and a similar calculation instituted. The results of thirty-three experiments, thus performed, showed, 1, that in no case was the weight increased during immersion; 2, that a little less was lost in the bath than in the air, but that this diminution varied much according to particular circumstances, especially according to the greater or less temperature of the water; 3, that, at a pressure of twenty-eight inches of mercury, the loss of weight in the water was, to that in the air, in the proportion of 6.5 to 17, provided the temperature of both media was not above 10° or 12° Reaumur; 4, that, under the same barometric pressure, the proportion is as 7.5 to 21.7, the temperature being from 15° to 18°, and, as 13 to 23, when the temperature was so high as 26° or 28° R.

Now, it is necessary to remember that the contact of water does not prevent the process of exhalation. The experiments of Edwards are referred to as conclusive on this point. He found that cold-blooded animals, when confined in air saturated with moisture, and at the same temperature as their bodies, and when, consequently, no perspiration by evaporation could take place, were yet found to lose weight; an effect solely ascribable to transudation. In man and other warm-blooded animals, when exposed to the same conditions, transudation takes place to such an extent that the sweat streams from all parts of the body. Thus, Delaroche and Berger found that air, very hot, and charged with extreme humidity, excited a more abundant transudation than dry air at a higher temperature. Now, if these facts are applied to the warm-bath, it will be seen that transudation is favoured, and that this will take place the more abundantly according as the temperature is more elevated. Thus, in his experiments on batrachians, Edwards found that, on comparing the total losses in the space of six hours at the temperature of 32° F. and those which took place at 104° F., they were nearly as 1 to 55. But does evaporation take place from the lungs during the use of the warm bath? Is the atmosphere so saturated with moisture that pulmonary evaporation ceases, as some have supposed? Dr. Madden, and apparently with justice, decides on the negative. In his joint memoir with Lavoisier, M. Séguin has stated that the proportion which the pulmonary bears to the general exhalation is as 7 to 18. In his first series of experiments, at a temperature of 60° F., the quantity lost in the bath was to that lost in the air as 6.5 to 17; showing that here the lungs alone were concerned, and, consequently, since cutaneous transpiration was proceeding as before, a quantity of moisture sufficient to supply this loss must have been absorbed. In the second series, at a temperature between 70° and 80° F., the result is still more favorable, the proportion being as 7.5 to 21.7; but, in the third, the loss was much greater, the proportion being as 13 to 23, the temperature being about 100° F.

These experiments are regarded by Dr. Madden, therefore, as conclusive evidence in favour of water having been absorbed. Whether this effect were produced by the skin or the lungs many may be inclined to doubt, and those who deny the existence of cutaneous absorption will at once attribute it to the latter. It is certain that the bronchial membrane absorbs some substances with great rapidity, e. g. turpentine, vapour of mercury, &c.: but it is contended that evidence is quite wanting in favour of the absorption of water by this membrane. The experiments which Dr. Madden has made tend to show that, if the lungs are capable of such absorption, the capability is very small, and very variable: and, he remarks, that, in thus gratuitously conferring this power upon the lungs, (and it must be confessed that evidence is wanting in its favour,) the difficulty remains of explaining why no such absorption occurred in the experiments of Séguin and others.

We have thus abridged the author's observations on Séguin's experiments, because they appear to render extremely doubtful the conclusions which have been drawn from these. We have but one remark to make on them; and that is, that those who seek for an *experimentum crucis* may object that, although cutaneous exudation takes place very rapidly in a heated moist atmosphere, it is not *therefore* to be inferred that it occurs equally in hot water. The subject probably admits of no closer demonstration, but, as the circumstances are not precisely similar, the objection may be made; and will be estimated of more or less importance according to individual views and bias. We do not regard it as necessary to allude to any more negative facts, such as those mentioned by Dr. Currie; and without quoting any of the experiments of Dr. Young, with which our readers are familiar, or those of Dr. Dill, we proceed to our author's own experiments.

"The balance which I employed was extremely sensible, vibrating perceptibly with a weight of a very few grains; and, in order to obtain the greatest possible accuracy in my results, I made use of the following plan. Having carefully noted the spot to which the extremity of the beam, when perfectly horizontal, pointed, this was taken as the centre of a scale, constructed with the greatest care, by adding weights alternately to the beam and the seat, and marking the respective points which the beam indicated. By this means I was enabled to measure with the greatest accuracy any weight not less than gr. x., but lower than this I did not attempt to go, nor do I think that it was necessary. The scale then being affixed to the wall, and the balance so arranged that the extremity of the beam pointed exactly to the horizontal line, I was accurately weighed and the same process was repeated in half an hour. Immediately afterwards, I entered the bath, and to avoid the most distant chance of fallacy from pulmonary absorption, my head was enveloped in an oiled-cloth bag, to which a long glass tube was attached, and passed out of window, so that I breathed the external air alone. After remaining immersed for the same length of time, I was carefully dried and again weighed. The results of twelve experiments thus performed, though not uniformly the same, were yet, upon the whole, extremely satisfactory. The whole of these bath experiments were performed in August and September 1836, the weather being for the most part fine, and the air of moderate temperature." (p. 58-9.)

We give one of Dr. Madden's experiments in detail, and adjoin a table of the whole.

"*Experiment* xi. At a quarter before seven A.M. having just risen, my weight was 111lb. + ℥j. + ʒiv. + ℈ij. In an hour and half, that is, at a quarter past eight, I had lost ℥ij. + ʒvj., giving an average expenditure of ʒvij. + ℈j. during the half hour. I then entered the bath at 84° F. and remained immersed during the space of thirty minutes. At the expiration of this period, I had *gained* ℥ss. Add to this, ʒiij. for pulmonary exhalation, and the whole increase, even upon the supposition that cutaneous transpiration was completely suspended, amounted to ʒvij." (p. 59.)

| Exp. | Date. | Time. | Bar. | Expenditure. | Temperature. | Gain. | Loss. |
|---|---|---|---|---|---|---|---|
| 1 | Aug. 20 | 8 A.M. | 29.63 | ʒvij. + ℈j. | 84° F. | ʒiv. | |
| 2 | ... 22 | 8 A.M. | 29.35 | ʒv. | 84°—88° F. | | ʒij. + ℈j. |
| 3 | ... 24 | 7½ A.M. | 29.96 | ʒvj. | 84° F. | ʒj.+℈j.+ gr.x. | |
| 4 | ... 25 | 7½ A.M. | 29.60 | ʒvj. + gr. x. | 84 ... | ʒj. | |
| 5 | ... 26 | 4 P.M. | 29.30 | ʒijss. | 88 ... | ʒj.+℈ij.+gr.x. | |
| 6 | Sept. 2 | 7 P.M. | 29.60 | ʒjxss. | 88 ... | ʒj. + ℈j. | |
| 7 | ... 5 | 6½ P.M. | 29.45 | not observed. | 94 ... | ʒj. | |
| 8 | ... 6 | 4 P.M. | 29.5 | ʒvij. | 98 ... | | ℥ij.+ʒij.+gr.x. |
| 9 | ... 7 | 6 P.M. | 29.8 | not observed. | 91 ... | ℈ij. | |
| 10 | ... 8 | 3½ P.M. | 29.65 | ʒvj. + ℈ij. | 92 ... | ℈ij. | |
| 11 | ... 9 | 7 P.M. | 29.8 | ℥j.+℈ij.+gr.x. | 90 ... | ʒjss. | |
| 12 | ... 10 | 4 P.M. | 29.9 | ʒvjss. | 90 ... | ʒv. + ℈j. | |

(p. 63.)

On the above analysis of his experiments, Dr. Madden remarks that it affords indubitable evidence in favour of cutaneous absorption; although this function appears to be exceedingly variable, and it is very difficult to discover any general laws by which it is regulated. We certainly can hit on no other explanation of the above facts, conducted as they appear to have been with great caution, and a knowledge and avoidance of those sources of error which have rendered the experiments of some previous enquirers of no utility in arriving at a safe judgment on the subject before us. Still it is very unsatisfactory to meet with such different results of similar experiments, conducted by different individuals. It is, no doubt, wrong to allow (admitting the credibility of the facts adduced, and admitting also the completeness of such facts,) positive conclusions to be affected by negative ones: at the same time it is remarkable, and we know not how to explain it, that, in ten out of twelve of Dr. Madden's experiments, there appears to have been actual increase of weight in the bath; whilst Séguin states that in no instance was the weight increased during immersion; and this remark applies to no less than thirty-three experiments, instituted with great care. These discrepancies are among the wonders that never cease: and similar ones meet us not only on this subject but in all physiological subjects which admit of doubt and experiment and difference of opinion. May they not be supposed to indicate that we are ignorant of some fundamental fact which may hereafter be discovered to the disturbance of much of our present knowledge, and which, perhaps, it is essential that we should be acquainted with, before attempting to explain them?

Chapter ii. is on *Absorption of Aqueous Vapour from the Atmosphere.* On this point it is not to be expected than any very satisfactory evidence can be at present obtained. For lovers of the marvellous this chapter contains many curious histories of the enormous disproportion between the quantities of urine and other fluids excreted, and the quantity of fluid taken into the body. It appears also that, in certain cases, thirst is allayed and life preserved by the moisture of the atmosphere alone; that there are recorded examples of the *actual increase of weight* which the body has been found to obtain through the medium of a moist atmosphere. "Thus De Gorter observed that under these circumstances, his body would absorb from two to six ounces during the night." But being somewhat incredulous of the virtues of jockeys we should hesitate to ascribe to cutaneous absorption the fact that "a jockey who had been for some time in training for a race—and who had been reduced to the proper weight, on the morning of the trial, being much oppressed with thirst, took a cup of tea, and shortly afterwards his weight was increased 6lb., so that he was incapacitated for riding." We do not doubt the fact, but we have some suspicions that the individual in question could have explained it without reference to cutaneous absorption from a moist atmosphere. Mr. Bell has remarked, that frogs kept in a moist situation, without having access to water in any form but vapour, have a constantly moist skin, and a water bag filled—an effect which can only be ascribed to absorption. There is in these cases evidence of absorption, though not of the surface from which such absorption takes place. Do Dr. Madden's reasonings on the matter make sufficient allowance for the fact that the lungs are a *vital* organ?

We pass over chapter iii., on *Absorption of Contagious Matters and Miasms.*

Chapter iv. is on the *Absorption of Medicinal Agents.* The first section is on the *Absorption of Solids;* viz. "powders, ointments, solid extracts, plasters, and poultices, both when simply applied to the common integuments and when administered as frictions; the latter being mentioned, not so much in support of the doctrine, as with a view to its practical application in the treatment of disease." We shall briefly notice some of the facts under this head. Kellie mentions the fact of salivation from wearing a mercurial plaster. Tartar emetic, applied as a powder by Séguin, was absorbed, and without producing its usual local effects; but it has been known, when rubbed upon the skin, to produce nausea and vomiting. Arsenic has produced violent inflammation, when employed to destroy lice. Some purgatives applied to the skin of the abdomen, in a solid form, have acted on the bowels. Haller states that pills placed upon the epigastrium have purged, and that the bowels have been opened from handling colocynth. A rhubarb poultice will purge children. Frictions with squill and digitalis have been said to effect diuresis. Some of the effects of opium, applied in a solid form, are well known: the same may be said of belladonna, tobacco, veratria, assafœtida, &c. —*Absorption of Fluids.* Salivation has been produced by the absorption of a solution of corrosive sublimate. After immersing his arm in a solution of hydriodate of potass, Dr. Madden has distinctly detected iodine in his urine. He also rubbed a solution of tartar emetic in water into his hands: this was followed by an unusual degree of languor and debility, which continued for some hours, and was accompanied by frequent though slight attacks of nausea, but the appetite was unimpaired. No local action was excited. Solutions of salts of lead have been known to be absorbed from the skin. In experiments on his own person, Dr. Madden has produced purgative effects by infusion of rhubarb, jalap, and gamboge. The experiments performed with turpentine, which have reference to the subject of cutaneous absorption, are often referred to. Dr. Young's experiments are well known. Klapp and Rousseau repeated

Young's experiments, but with a contrary result. The following is our author's experience on this point: With his head enclosed in an oiled cloth bag, to which a long tube was attached, and passed out of the window, his arm being luted into a large glass jar, he had some turpentine poured upon it, and the jar accurately stoppered. The urine acquired a distinctly violet odour. The experiment was repeated four times, with equally satisfactory results. The effects of the external application of solutions of opium are well known; those produced by belladonna and tobacco are equally unequivocal. Dr. Madden has not been able to produce the physiological actions of nux vomica, by applying solutions of it to the skin of animals. The following is one of his experiments:

> "At ten minutes before eleven A.M., I dropped half a drachm of the oil (of bitter almonds) into the axillæ of a pigeon, and then introduced it into a jar, closed at the mouth with oiled silk, through which its head alone protruded, and was carefully luted. At 11 + 5′, the breathing became accelerated. In two minutes more, the bird vomited. At 11 + 12′, respiration was very laborious, and became gradually worse until 11 + 23′, when a fit of convulsions came on. After this, the breathing grew slower and slower, and death took place at ten minutes past twelve." (p. 122.)

In several experiments performed by Dr. Madden, he was unable to detect ferrocyanate of potass in his urine: but, although respecting some of the fluids mentioned in the above section, there may be a doubt whether their action was subsequent to cutaneous absorption or no, the instances of turpentine and iodine appear to justify no other inference.

*Absorption of Gases and Vapours.* Our present limits do not allow us to enter upon the disputed subject of cutaneous respiration in man. The contradictory results of experiments thereupon is sadly unsatisfactory. The following is an experiment of Collard de Martigny on the action of carbonic acid on the surface:

> "He placed himself entirely under the cloth which covered a deep tub, partly filled with fermenting grapes; and, his nostrils being accurately closed, he breathed through a long tube which communicated with the external air. 'In about five minutes,' says he, 'I felt a slight weight in the head and troubled vision; in eight minutes, considerable pain in the temples and under the orbit, ringing of the ears, and vertigo; in ten minutes, the same symptoms remained, with general weakness, and the heart's action became slightly accelerated; in twelve minutes, all these were aggravated, respiration became slow, a vague and indefinable terror seized my senses; and, finally, at the twenty-ninth minute, the weakness and torpor were so pronounced that the tube by which I breathed fell from me, and I could scarcely leave the spot.'" (p. 131.)

Dr. Madden found no effect produced on a pigeon, the body of which was confined in carbonic acid gas: but the action of sulphuretted hydrogen gas was very rapid upon a rabbit confined in it, so that it could not come in contact with the lungs. The anus of the animal was covered with sticking plaster. The rabbit died within twenty minutes. A solution of acetate of lead, poured beneath the skin and into the cavity of the peritoneum, gave distinct traces of the presence of this gas. The physiological action of mercury is capable of being produced by this metal in a state of vapour. Mr. Abernethy appears to have had a confidence in its efficacy which subsequent experience has not fully justified. The evidence in favour of the absorption of gaseous substances by the skin Dr. Madden does not consider as quite so satisfactory as that which he has adduced in the case of liquids.

Our Tenth Number contained an article on Endermic Absorption. It renders unnecessary any notice of Dr. Madden's chapter on the subject; and, indeed, there are other details which we have passed over, having already noticed them in that paper. From what has been previously said concerning the anatomy of the cutaneous system, it may be inferred that our author has nothing very satisfactory to suggest as to the agents by which absorption is effected. Without stating it as a decided opinion, he regards the first step in this process to be endosmosis. But there is so little that is satisfying in physiological speculation, that we are willing to give room for more important subjects.

We would fain hope that, in the disappointing path of practical medicine, our author may find that he has not over-estimated the advantages which cutaneous absorption is about to afford him. Commencing, as he has done, the theoretical part of his subject with such considerable care and such meritorious research, and being, as it were, a partisan (though an honest one) of the system of which he has shown himself so able an advocate, we shall be very glad to see a careful and unprejudiced detail of the effects produced by medicinal applications to the skin, in cases of disease, observed in the course of his own practice. It is a comparatively easy task to collect from a host of authors (of whose power, or even will, to observe correctly we almost require to be satisfied before we yield much credit to their recorded observations,) a number of cases illustrative of the benefit of this or that remedy in various forms of disease; and such an array of triumphs sometimes induces the forgetfulness that there may have been a far greater number of mishaps. That there is an advantage in the application of medicines to the surface, in various ways and forms, appears to be a growing opinion; and the facts collected by Dr. Madden, proving medicinal action through the skin, together with the additional evidence which he has afforded of the absorption of substances under various forms from it, render his essay well worthy of perusal, both by the physiologist and medical practitioner.

---

